# Supplementary material for: Formation of an expanding memory representation in the hippocampus
Source: Nat Neurosci. 2025 Jun 4;28(7):1510–8. doi: 10.1038/s41593-025-01986-3 (PMC12229897; doi:10.1038/s41593-025-01986-3)
Supplement: Supplementary file 1 — Reporting Summary [file 41593_2025_1986_MOESM1_ESM.pdf]

nature portfolio

Corresponding author(s): Jeffrey Magee

Last updated by author(s): Mar 20, 2025

Reporting Summary

Nature Portfolio wishes to improve the reproducibility of the work that we publish. This form provides structure for consistency and transparency in reporting. For further information on Nature Portfolio policies, see our Editorial Policies and the Editorial Policy Checklist.

Statistics

For all statistical analyses, confirm that the following items are present in the figure legend, table legend, main text, or Methods section.

n/a	Confirmed	
☐	☒	The exact sample size (*n*) for each experimental group/condition, given as a discrete number and unit of measurement
☐	☒	A statement on whether measurements were taken from distinct samples or whether the same sample was measured repeatedly
☐	☒	The statistical test(s) used AND whether they are one- or two-sided<br>*Only common tests should be described solely by name; describe more complex techniques in the Methods section.*
☐	☒	A description of all covariates tested
☐	☒	A description of any assumptions or corrections, such as tests of normality and adjustment for multiple comparisons
☐	☒	A full description of the statistical parameters including central tendency (e.g. means) or other basic estimates (e.g. regression coefficient) AND variation (e.g. standard deviation) or associated estimates of uncertainty (e.g. confidence intervals)
☐	☒	For null hypothesis testing, the test statistic (e.g. F, t, r) with confidence intervals, effect sizes, degrees of freedom and P value noted<br>*Give P values as exact values whenever suitable.*
☒	☐	For Bayesian analysis, information on the choice of priors and Markov chain Monte Carlo settings
☒	☐	For hierarchical and complex designs, identification of the appropriate level for tests and full reporting of outcomes
☐	☒	Estimates of effect sizes (e.g. Cohen's *d*, Pearson's *r*), indicating how they were calculated

Our web collection on statistics for biologists contains articles on many of the points above.

Software and code

Policy information about availability of computer code

Data collection	The Ca2+ imaging data was acquired using a National Instruments PCI system, controlled by ScanImage(R2020, Vidrio). The behavioral data was acquired via a PCIe-6343, X series DAQ system (National Instruments) using WaveSrufer software (wavesurfer.janelia.org). The behavioral system was controlled by a BPOD module (Sanworks LLC) that interfaced with arduino-based microcontrollers using custom written Arduino and Matlab code.
Data analysis	Acquired Calcium imaging data was motion corrected using Suite2P (Python version, https://github.com/MouseLand/suite2p). Image segmentation was manually performed using ImageJ(version 2.9.0), and calcium traces extracted using custom code in Python(scipy.ndimage). Further analysis of CA1 neuronal activity was performed using custom functions written in MATLAB (Version 2018a, 2020a). The modeling was performed in IGOR 8.04. The code used to analyze the experimental data and modeling will be available on FigShare at 10.6084/m9.figshare.28628939

For manuscripts utilizing custom algorithms or software that are central to the research but not yet described in published literature, software must be made available to editors and reviewers. We strongly encourage code deposition in a community repository (e.g. GitHub). See the Nature Portfolio guidelines for submitting code & software for further information.

Data

Policy information about availability of data

All manuscripts must include a data availability statement. This statement should provide the following information, where applicable:

- Accession codes, unique identifiers, or web links for publicly available datasets
- A description of any restrictions on data availability
- For clinical datasets or third party data, please ensure that the statement adheres to our policy

Raw Data will be available on FigShare at 10.6084/m9.figshare.28628939 after publication

Research involving human participants, their data, or biological material

Policy information about studies with human participants or human data. See also policy information about sex, gender (identity/presentation), and sexual orientation and race, ethnicity and racism.

Reporting on sex and gender	N/A
Reporting on race, ethnicity, or other socially relevant groupings	N/A
Population characteristics	N/A
Recruitment	N/A
Ethics oversight	N/A

Note that full information on the approval of the study protocol must also be provided in the manuscript.

Field-specific reporting

Please select the one below that is the best fit for your research. If you are not sure, read the appropriate sections before making your selection.

☒ Life sciences ☐ Behavioural & social sciences ☐ Ecological, evolutionary & environmental sciences

For a reference copy of the document with all sections, see nature.com/documents/nr-reporting-summary-flat.pdf

Life sciences study design

All studies must disclose on these points even when the disclosure is negative.

Sample size	No statistical methods were used to predetermine sample sizes, but our sample sizes are similar to those reported in previous publications29,30,64 using a similar behavioral task and by the expected number of active neurons that can be imaged with the two-photon microscope in awake behaving mice
Data exclusions	Animals were excluded from further analysis for three reasons: 1) extensive z-motion that precluded imaging of the same population throughout the session. 2) Significant inter-day shift in imaging FOV that barred imaging of the same neurons on subsequent days. 3) an animal that failed to run 4 epochs (~60 laps) in a recording session of 60 minutes on any day of longitudinal imaging.
Replication	All experiments were performed independently. Our main findings are maintained across all animals (Extended Data Fig. 4), serving as inherent biological replicates.
Randomization	Differential experimental groups were not established for the primary findings, so randomization was not needed.
Blinding	Differential experimental groups were not established for the primary findings, so randomization was not needed.

Reporting for specific materials, systems and methods

We require information from authors about some types of materials, experimental systems and methods used in many studies. Here, indicate whether each material, system or method listed is relevant to your study. If you are not sure if a list item applies to your research, read the appropriate section before selecting a response.

Materials & experimental systems

n/a	Involved in the study
☒	☐ Antibodies
☒	☐ Eukaryotic cell lines
☒	☐ Palaeontology and archaeology
☐	☒ Animals and other organisms
☒	☐ Clinical data
☒	☐ Dual use research of concern
☒	☐ Plants

Methods

n/a	Involved in the study
☒	☐ ChIP-seq
☒	☐ Flow cytometry
☒	☐ MRI-based neuroimaging

Animals and other research organisms

Policy information about studies involving animals; ARRIVE guidelines recommended for reporting animal research, and Sex and Gender in Research

Laboratory animals	All experiments were performed in GP5.17 mice (>10 weeks, Jackson Laboratory, stock no. 025393).
Wild animals	The study does not involve wild animals
Reporting on sex	Mice of either sex were used for this study.
Field-collected samples	No Field-collected samples were used for this study.
Ethics oversight	All experiments were performed according to methods approved by Baylor College of Medicine's (Protocol AN-7734) IACUC committee.

Note that full information on the approval of the study protocol must also be provided in the manuscript.

Plants

Seed stocks	N/A
Novel plant genotypes	N/A
Authentication	N/A
